# Swelling-Activated Anion Channels Are Essential for Volume Regulation of Mouse Thymocytes

**DOI:** 10.3390/ijms12129125

**Published:** 2011-12-08

**Authors:** Ranokhon S. Kurbannazarova, Svetlana V. Bessonova, Yasunobu Okada, Ravshan Z. Sabirov

**Affiliations:** 1 Department of Cell Physiology, National Institute for Physiological Sciences, Okazaki 444-8585, Japan; E-Mails: psharapovna@yahoo.com (R.S.K.); bessosvetlana@mail.ru (S.V.B.); sabirov@nips.ac.jp (R.Z.S.); 2 Laboratory of Molecular Physiology, Institute of Physiology and Biophysics, Acad. Sci. RUz, Tashkent 100095, Niyazova 1, Uzbekistan; 3 Department of Biophysics, National University, Tashkent 100174, Vuzgorodok, Uzbekistan

**Keywords:** thymocytes, volume regulation, anion channels, phloretin, DIOA

## Abstract

Channel-mediated trans-membrane chloride movement is a key process in the active cell volume regulation under osmotic stress in most cells. However, thymocytes were hypothesized to regulate their volume by activating a coupled K-Cl cotransport mechanism. Under the patch-clamp, we found that osmotic swelling activates two types of macroscopic anion conductance with different voltage-dependence and pharmacology. At the single-channel level, we identified two types of events: one corresponded to the maxi-anion channel, and the other one had characteristics of the volume-sensitive outwardly rectifying (VSOR) chloride channel of intermediate conductance. A VSOR inhibitor, phloretin, significantly suppressed both macroscopic VSOR-type conductance and single-channel activity of intermediate amplitude. The maxi-anion channel activity was largely suppressed by Gd^3+^ ions but not by phloretin. Surprisingly, [(dihydroindenyl)oxy] alkanoic acid (DIOA), a known antagonist of K-Cl cotransporter, was found to significantly suppress the activity of the VSOR-type single-channel events with no effect on the maxi-anion channels at 10 μM. The regulatory volume decrease (RVD) phase of cellular response to hypotonicity was mildly suppressed by Gd^3+^ ions and was completely abolished by phloretin suggesting a major impact of the VSOR chloride channel and modulatory role of the maxi-anion channel. The inhibitory effect of DIOA was also strong, and, most likely, it occurred via blocking the VSOR Cl^−^ channels.

## 1. Introduction

All living cells constantly experience osmotic challenges due to fluctuations in the concentration of the osmotically active constituents of the cytosol caused by intensive metabolic activity and of the extracellular fluids because of the intensive gas, water, nonelectrolyte and ion exchange at the epithelial surfaces along the airways, in kidney, brain, primary and secondary lymphoid organs. In order to survive, the cells must actively regulate their volume and maintain it near the level optimal for normal life [1–3]. Under hypoosmotic stress, most of the cells first swell and then actively restore their volume by extruding osmolytes (K^+^, Cl^−^, taurine and some metabolites) from the cytoplasm, a process called Regulatory Volume Decrease (RVD). Recent studies suggest that the fully functional RVD machinery is strictly required for cell proliferation and apoptotic cell death [1,4–9].

Bone marrow-derived precursor cells rapidly proliferate in thymus forming a pool of immature thymic lymphocytes, thymocytes. Most of them eventually die by apoptosis during positive selection for T-cell receptor and negative selection for auto-reactive cells [10,11]. Thymocytes possess a potent RVD system and effectively restore their volume after osmotic swelling [12–15]. We previously showed that water permeability of the thymocyte plasma membrane is required for the post-swelling volume decrease [13]. However, the mechanism of the osmolyte efflux from thymocytes during RVD remains controversial. Arrazola *et al.* [12] and Soler *et al.* [15] found that osmotic swelling-induced fluxes of K^+^ and Cl^−^ were tightly coupled and were efficiently inhibited by a selective blocker of the K-Cl cotransporter, [(dihydroindenyl)oxy] alkanoic acid (DIOA). However, in our previous pharmacological study, the RVD process in rat thymocytes was completely abolished by blockers of potassium channels and swelling-activated anion channels [14] suggesting involvement of ion channels rather than the coupled K-Cl cotransporter in thymocyte volume regulation under hypotonicity. Here, we provide direct evidence that cell swelling activates two types of the volume-regulated anion channels, the volume-sensitive outwardly rectifying (VSOR) Cl^−^ channel and the maxi-anion channel; and only the VSOR channel is chiefly responsible for the anionic efflux during RVD in thymocytes and is sensitive to DIOA.

## 2. Results and Discussion

### 2.1. Whole-Cell Anion Currents Activated in Mouse Thymocytes in Response to Osmotic Cell Swelling

In our preliminary experiments, we attempted to measure the macroscopic swelling-induced whole-cell currents using a conventional method of decreasing the extracellular osmolality. However, in experiments with isotonic pipette solutions, the cells spontaneously swelled due to oncotic pressure gradient, whereas even slight hypotonicity of pipette solution used to prevent spontaneous cell swelling [16] led to visible shrinkage of thymocytes. In most cases, subsequent application of hypotonicity deteriorated the cells and did not induce reproducible swelling-activated whole-cell currents. In contrast, when we induced cell swelling by using hypertonic pipette solutions, the whole-cell configuration was more stable, and reproducible macroscopic currents could be recorded. Therefore, in the present study we induced cell swelling by using a hypertonic pipette solution made by adding mannitol.

**Figure 1:**
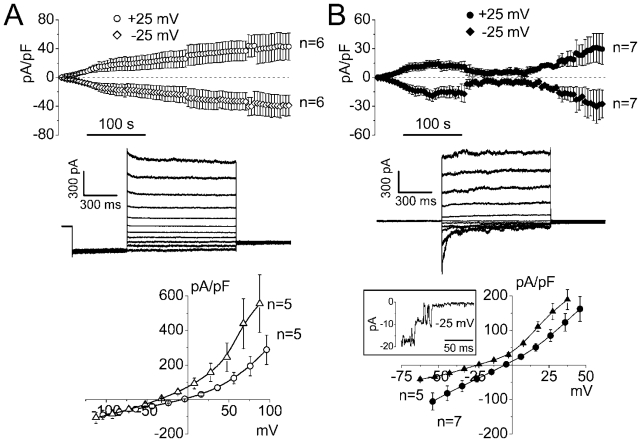
Two types of whole-cell macroscopic currents activated by cell swelling in mouse thymocytes. The pipette solution contained either 3 mM ATP (**A**) or no ATP (**B**). (Top panel) Time course of whole-cell current activation in response to cell swelling. Currents were elicited by application of alternating pulses from 0 to ±25 mV (every 5 s). (Middle panel) Representative traces of current responses recorded after maximal current activation. In A, the holding potential was 0 mV; after a pre-pulse to −100 mV (500 ms), currents were elicited by application of step-pulses (1000 ms) from −100 to +100 mV in 20-mV increments. In B, the holding potential was 0 mV; currents were elicited by application of step pulses (1000 ms) from −50 to +50 mV in 10-mV increments. (Bottom panel) Instantaneous current-to-voltage relationships measured at the beginning of test pulses after maximal current activation; pipette solution contained 125 mM Cl^−^ (circles) or 25 mM Cl^−^ (triangles). The inset on the bottom panel in B shows single maxi-anion channel-like current fluctuations seen in whole-cell recordings.

Immediately after rupturing the membrane patch and attaining the whole-cell configuration, the whole-cell currents were low with a current density of 0.04 ± 0.01 pA/pF and 0.005 ± 0.002 pA/pF at +25 mV and −25 mV, respectively. The whole-cell currents gradually increased upon cell swelling and reached values of 40.2 ± 19.9 pA/pF and −38.3 ± 15.7 pA/pF at +25 mV and −25 mV, respectively, after 6 min (Figure 1A, top panel). Longer swelling usually resulted in deterioration of the cells. The current responses to step pulses exhibited time-dependent inactivation at positive potentials larger than +80 mV (Figure 1A, middle traces). As shown in Figure 1A (bottom panel), the current-voltage relationship showed outward rectification and reversed at −4.4 ± 1.7 mV (open circles). The reversal potential shifted to the value of −33.7 ± 1.6 mV upon reduction of the pipette Cl^−^ concentration from 125 mM to 25 mM by equimolar replacement of Cl^−^ with aspartate^−^ (open triangles) indicating an anion selectivity of the whole-cell macroscopic conductance with *P*_Asp_/*P*_Cl_ = 0.08 ± 0.02. These characteristics are typical of VSOR Cl^−^ current [8,9,17].

In addition to the VSOR Cl^−^ channel current, cell swelling is known to activate another type of anionic current, which is characterized by less or no rectification and voltage-dependent inactivation at potentials of about ±20–30 mV. The channel mediating this current has very large conductance of about 300–400 pS and termed maxi-anion channel [18]. High intracellular ATP is known to favor activation of VSOR Cl^−^ channels, whereas metabolic stress is favorable for the maxi-anion channels [19–21]. In osmotically swollen thymocytes, omitting ATP from the pipette solution led to a biphasic activation of the macroscopic whole-cell currents. Membrane currents increased first to a level of 14.2 ± 4.3 pA/pF and −17.2 ± 5.4 pA/pF at +25 mV and −25 mV, respectively. Then the current decreased to about 25–35% of the peak value and increased again, reaching the level of 26.1 ± 13.4 pA/pF and −25.4 ± 15.4 pA/pF at +25 mV and −25 mV, respectively, after 6 min of swelling (Figure 1B, top panel). The declining phase of the whole-cell current in Figure 1B was likely induced by washing out the residual ATP from the cytosol, but not because of the regulatory volume decrease which is impossible in the whole-cell mode of patch-clamp. Longer swelling usually resulted in further current increase, but the whole-cell configuration was less stable. The current responses to step pulses exhibited time-dependent inactivation at negative potentials larger than −20 mV (Figure 1B, middle traces). The current-voltage relationships showed a slight outward rectification and reversed at −4.7 ± 0.8 mV (Figure 1B, bottom panel: filled circles). The reversal potential shifted to the value of −22.4 ± 1.5 mV upon reduction of the pipette Cl^−^ concentration from 125 mM to 25 mM (filled triangles) indicating an anion selectivity of the whole-cell macroscopic conductance with *P*_Asp_/*P*_Cl_ = 0.29 ± 0.04. These characteristics are different from the VSOR Cl^−^ current and resemble the maxi-anion channel-type of macroscopic conductance observed in mammary C127 cells [22] and cultured neonatal cardiomyocytes [19]. This suggestion was further supported by the fact that at the initial stage of the second rising phase of the macroscopic whole-cell currents (at around 4 min) we often observed stepwise current fluctuations of about 8–10 pA at −25 mV characteristic to the maxi-anion channel. An example of such recording is shown on the inset of Figure 1B (bottom panel).

**Figure 2:**
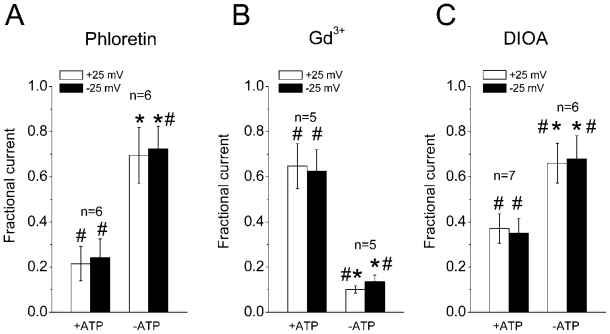
Pharmacological profiles of whole-cell currents activated by an osmotic challenge in thymocytes. Effects of 200 μM phloretin (**A**), 50 μM Gd^3+^ (**B**) and 10 μM DIOA (**C**) on mean currents recorded at +25 mV (open columns) and −25 mV (filled columns). Data are normalized to the mean current measured before application of drugs. Each column represents the mean ± SEM (vertical bar). # Significantly different from the control current without drug at *P* < 0.05. * Significantly different from the current measured with ATP-containing pipette solution at *P* < 0.05.

As shown in Figure 2A, the swelling-induced whole-cell current recorded with ATP-containing pipette solution was potently inhibited by a bisphenol, phloretin, which allows discrimination of the VSOR Cl^−^ currents from Ca^2+^-activated and cAMP-activated Cl^−^ currents [23]. Effect of phloretin was significantly less in the absence of ATP in the pipette solution (Figure 2A) indicating smaller contribution of VSOR Cl^−^ channels to the macroscopic conductance measured in the absence of ATP. In contrast, the swelling-induced whole-cell current was significantly more affected by Gd^3+^ ions in the absence of ATP in the pipette solution compared to the current measured with ATP-containing pipettes (Figure 2B). Since among anion channels Gd^3+^ blocks only the maxi-anion channel, we suggest that the latter constitutes a major part of the conductance activated in the absence of intracellular ATP. Surprisingly, a commonly used K-Cl cotransporter inhibitor, DIOA, also suppressed the swelling-induced macroscopic currents at 10 μM (Figure 2C) with efficiency more pronounced in the presence of ATP in the pipettes. Since the K-Cl cotransport is electroneutral, we supposed that swelling-induced anion channels might be sensitive to this drug. A similar effect was reported earlier for nonpigmented ciliary epithelium [24].

### 2.2. Single Anion Channel Currents Activated in Osmotically Swollen Mouse Thymocytes

**Figure 3:**
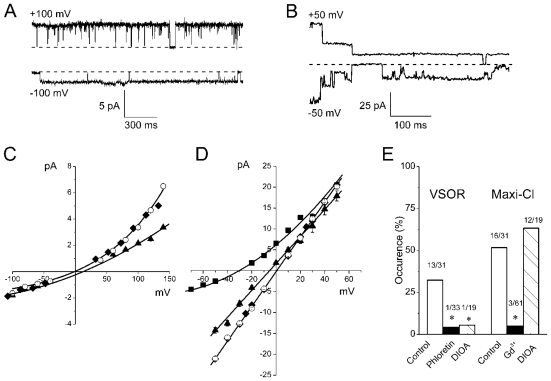
Two types of microscopic currents activated in osmotically swollen thymocytes. (**A**) Single VSOR anion channel activity. (**B**) Single maxi-anion channel activity. Pipette: 100 mM TEACl-pipette solution; bath: hypotonic high-K^+^ solution. Dashed lines correspond to zero current level. (**C**) I-V relationship for the single-channel events similar to those shown in (**A**). Unitary currents were recorded in the cell-attached mode with standard 100 mM CsCl-pipette solution (open circles), 100 mM TEACl-pipette solution (closed diamonds) and 30 mM CsCl-pipette solution (filled triangles). Each data point represents the mean ± SEM of 5 to 29 measurements from 8–14 different patches. (**D**) I-V relationship for the single-channel events similar to those shown in (**B**). Unitary currents were recorded in the cell-attached mode with 100 mM TEACl-pipette solution and hypotonic high-K^+^ solution in the bath (filled triangles), or in inside-out mode with Ringer solution in the pipette and in the bath (open circles). Filled diamonds and filled squares: 135 mM NaCl in the bath solution was replaced with equimolar TEACl and Na-glutamate, respectively (inside-out, pipette filled with Ringer solution). Each data point represents the mean ± SEM of 5 to 30 measurements from 7–10 different patches. (**E**) Effects of phloretin (200 μM), DIOA (10 μM) and Gd^3+^ ions (50 μM) on the occurrence of the VSOR-type and maxi-anion-type channels in the on-cell mode (the drugs added to the pipette solution). On the top of the bars: number of channel-containing patches/total number of patches. * Significantly different from the control value at *P* < 0.05.

Cell-attached patches exhibited a very low level of single-channel activity when giga-seals were formed before applying osmotic stress. This is consistent with the previous observation that volume-sensitive anion channels can be monitored only in membrane patches formed after cell swelling [17]. When cells were swollen in hypotonic high-K^+^ solution prior to seal formation for approx. 5–10 min, a considerably higher level of single-channel activity was observed in the on-cell mode. We mainly observed two groups of single-channel events (Figure 3A,B). One group displayed an intermediate single-channel amplitude of 4.31 ± 0.12 pA (*n* = 13) at +100 mV and −1.88 ± 0.08 pA (*n* = 20) at −100 mV (Figure 3A). The single-channel I-V relationship for this group displayed profound outward rectification and was insensitive to the replacement of TEA^+^ with Cs^+^ in the pipette solution (Figure 3C). Reducing the pipette CsCl concentration from 100 to 30 mM reduced the channel amplitude (measured at +140 mV) to 52 ± 5% and caused a positive shift of the reversal potential of about 11 ± 5 mV (Figure 3C). These results are consistent with anionic selectivity of this channel. Thus, outward rectification, intermediate conductance and anion selectivity reproduce the phenotype of the VSOR anion channels observed earlier in Intestine 407 cells [17,25–27] and other cell types [28–32].

In the next series of experiments, we monitored the single-channel activity on osmotically swollen cells in the cell-attached mode in the presence of inhibitors of volume-sensitive anion channels and K-Cl cotransport in the pipette solution. The channel occurrence rather than the channel open probability is used to evaluate the channel activity, since in many instances the membrane patches contained both types of the channels. Consistent with previous observations, the VSOR-type single-channel events were effectively inhibited by phloretin, whereas maxi-anion channel-type events were blocked by Gd^3+^ ions (Figure 3E). A K-Cl cotransport inhibitor, DIOA, largely eliminated the activity of VSOR-type channels, but not maxi-anion channels (Figure 3E).

The second type of single-channel events displayed large single-channel amplitude of 17.9 ± 1.2 pA (*n* = 7) at +50 mM and −14.3 ± 0.7 pA (*n* = 20) at −50 mV and linear I-V relationship (Figure 3D). The slope conductance was 322 ± 7.8 pS in the on-cell mode with TEA-Cl pipette solution and hypotonic high-K^+^ solution in the bath and 418 ± 3.2 pS in excised inside-out mode with symmetrical Ringer solution in the bath and in the pipette. In inside-out experiments, the single-channel I-V relationship was insensitive to the replacement of Na^+^ with TEA^+^ in the bath Ringer solution (Figure 3D), whereas reducing Cl^−^ concentration from 146 to 11 mM by replacing NaCl with equimolar Na-glutamate shifted the reversal potential to a value of −30.4 ± 0.3 mV (Figure 3D). This result indicates that the channel is anion-selective with a permeability ratio of glutamate^−^ to Cl^−^ of 0.211 ± 0.003. These results are consistent with anionic selectivity of this channel. Thus, linear I-V relationship, large single-channel conductance and anion selectivity reproduce the phenotype of the maxi-anion channels observed earlier in other cell types [19–22,33–36].

### 2.3. Sensitivity of the Regulatory Volume Decrease to Blockers for VSOR Cl^−^ Channel and K-Cl Cotransport

**Figure 4:**
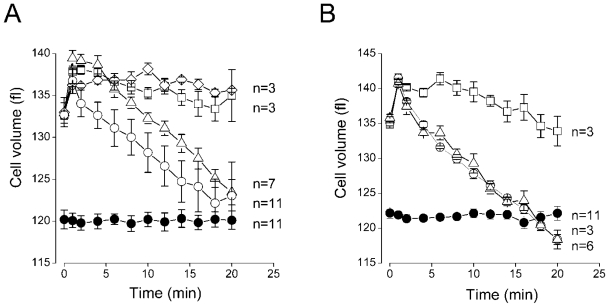
Effects of inhibitors of swelling-activated anion channels and K-Cl cotransporter on volume regulation of mouse thymocytes. (**A**) Time courses of cell volume change in isotonic conditions (filled circles) and in hypotonic conditions in the absence (open circles) and presence of 50 μM Gd^3+^ (open triangles), 50 μM phloretin (open squares) and 250 μM glibenclamide (open diamonds). (**B**) Time courses of cell volume change in isotonic conditions (filled circles) and in hypotonic conditions in the absence (open circles) and presence of 1 μM and 10 μM DIOA (open triangles and open squares, respectively). The cell volume was measured by Coulter-type cell sizing method.

When placed in the isotonic solution (290 mosmol/kg-H_2_O), the volume of murine thymocytes was stable at a level of 120.2 ± 1.5 fl (*n* = 11) for a period of 20 min (Figure 4A, filled circles). Under hypotonicity (69% osmolality), the cells first swelled to the maximal volume of 136.8 ± 1.5 fl (*n* = 11) within app. 2 min and then restored their volume down to the nearly initial value within app. 18 min (Figure 4A, open circles). Thus, the murine thymocytes effectively regulated their volume in response to the hypoosmotic stress displaying robust RVD. A VSOR Cl^−^ channel blocker, phloretin, effectively suppressed the RVD process at 50 μM (Figure 4A, open squares), whereas 50 μM Gd^3+^ ions were much less effective (Figure 4A, open triangles). These results suggest that the VSOR Cl^−^ channel plays a major role in the RVD of thymocytes with minor modulatory contribution of the maxi-anion channel. In order to confirm the leading role of the VSOR Cl^−^ channel in thymic RVD, we tested another effective VSOR inhibitor, glibenclamide [37]. This drug was as effective as phloretin and completely abolished the thymocyte RVD at 250 μM (Figure 4A, open diamonds). DIOA was ineffective at 1 μM, but strongly inhibited the RVD of thymocytes at the concentration of 10 μM (Figure 4B). Data of Figure 2 and Figure 3 suggest that these effects are largely due to the inhibition of the VSOR-type of the swelling-activated chloride channels. The earlier observation that the swelling-induced anion conductance in nonpigmented ciliary epithelium is also suppressed by DIOA at 50–100 μM [24] suggests that not only thymic VSOR but the VSOR-type conductance in other types of cells might be sensitive to this reportedly selective K-Cl cotransporter inhibitor.

## 3. Experimental Section

### 3.1. Solutions

The standard Ringer solution contained (mM): 135 NaCl, 5 KCl, 2 CaCl_2_, 1 MgCl_2_, 5 Na-HEPES, 6 HEPES, and 5 glucose (pH 7.4, 290 mosmol/kg-H_2_O). The pipette solution for whole-cell experiments contained (mM): 125 CsCl, 2 CaCl_2_, 1 MgCl_2_, 3 Na_2_ATP, 5 HEPES (pH 7.4 adjusted with CsOH), and 10 EGTA (pCa 7.65; 275 mosmol/kg-H_2_O). In some experiments Na_2_ATP was omitted when indicated. Hypertonic pipette solution (320 mosmol/kg-H_2_O) was made by adding 50 mM mannitol. For measurements of glutamate permeability of maxi-anion channels, the low-Cl^−^ bath solution was prepared by replacing 135 mM NaCl in standard Ringer solution with 135 mM Na-glutamate. For measurements of aspartate permeability of the VSOR Cl^−^ channels, the low-Cl^−^ pipette solution was prepared by replacing 100 mM CsCl in the whole-cell pipette solution with 100 mM Cs-aspartate. In on-cell single VSOR Cl^−^ and maxi-anion channel experiments, cells were bathed in hypotonic high-K^+^ solution containing (in mM): 100 KCl, 1 MgCl_2_, 2 CaCl_2_, 5 Na-HEPES, 6 HEPES, and 5 glucose (pH 7.4, 218 ± 4 mosmol/kg-H_2_O). The pipette was filled with a solution containing (in mM): 100 TEACl, 1 MgCl_2_, 2 CaCl_2_, and 5 HEPES (pH 7.4 adjusted with CsOH, 208 ± 3 mosmol/kg-H_2_O). In some experiments, 100 mM TEACl in pipette solution was replaced with 100 mM CsCl or 30 mM CsCl.

For cell volume measurements, the isotonic solution contained (mM): 90 NaCl, 5 KCl, 2 CaCl_2_, 1 MgCl_2_, 5 Na-HEPES, 6 HEPES, 5 glucose and 90 mM mannitol (pH 7.4, 290 mosmol/kg-H_2_O). The hypotonic (200 mosmol/kg-H_2_O, 69% hypotonicity) solution was made from the isotonic solution by omitting mannitol.

GdCl_3_ was stored as a 50 mM stock solution in water and added directly to Ringer solution immediately before each experiment. Phloretin and DIOA were purchased from Sigma-Aldrich and were added to Ringer solution immediately before use from stock solutions in DMSO. DMSO did not have any effect, when added alone at a concentration less than 0.1%.

### 3.2. Cells

The experimental protocol was approved in advance by the Ethics Review Committee for Animal Experimentation of the National Institute for Physiological Sciences. Cell isolation was performed as described previously [13,14,38]. Briefly, the 6–8 weeks old mice were anaesthetized with halothane and painlessly euthanized by cervical dislocation; the thymi were dissected and carefully washed with an ice-cold Ringer solution. The thymi were then minced using fine forceps and passed through a 100 μm-nylon mesh. The suspension was centrifuged at 1000 g for 5 min, the pellet was washed two times with Ringer solution and resuspended in this medium at a final concentration of 100 × 10^6^ cell/mL. The suspension was kept on ice for ≤ 5 h and contained no more than 5% of damaged cells as assayed by trypan blue exclusion.

### 3.3. Cell Volume Measurements

The mean cell volume was measured at room temperature using a Coulter-type cell sizing apparatus (CDA-500, Sysmex, Kobe, Japan), as reported previously [39].

### 3.4. Electrophysiology

Patch electrodes were fabricated from borosilicate glass capillaries using a laser micropipette puller (P-2000, Sutter Instrument, Novato, CA) and had a tip resistance of 3–5 MΩ when filled with pipette solution. Fast and slow capacitative transients were routinely compensated for. For whole-cell recordings, the access resistance did not exceed 10 MΩ and was always compensated for by 80%. Membrane currents were measured with an EPC-9 patch-clamp system (Heka-Electronics, Lambrecht/Pfalz, Germany). The membrane potential was controlled by shifting the pipette potential (*Vp*) and is reported as *Vp* for whole-cell recordings and −*Vp* for cell-attached and inside-out recordings. Currents were filtered at 1 kHz and sampled at 5–10 kHz. Data acquisition and analysis were done using Pulse + PulseFit (Heka-Electronics). Whenever the bath Cl^−^ concentration was altered, a salt bridge containing 3 M KCl in 2% agarose was used to minimize variations of the bath electrode potential. Liquid junction potentials were calculated using pCLAMP 8.1 (Axon Instruments, Foster, CA) algorithms and were corrected off-line when appropriate. All experiments were performed at room temperature (23–25 °C).

### 3.5. Data Analysis

Single-channel amplitudes were measured by manually placing a cursor at the open and closed channel levels. The reversal potentials were calculated by fitting I-V curves to a second-order polynomial [40]. Data were analyzed using Origin, versions 5–7 (OriginLab Corporation, Northampton, MA). Pooled data are given as means ± SEM of *n* observations. Comparisons between two experimental groups were made using the unpaired Student’s *t* test. For statistical analysis of the single channel occurrence (data of Figure 3E), the occurrence probability parameter (*op*) was set to *op* = 1 for patches displaying the channel activity and to *op* = 0 for patches lacking the channel activity; the two groups were then compared by the unpaired *t* test. Differences were considered to be statistically significant at *P* < 0.05.

## 4. Conclusions

Different pathways are known to be involved in the osmolyte efflux during RVD in different types of cells. Osmotic swelling-induced K^+^ and Cl^−^ extrusion may occur via three possible mechanisms [1–3,41]: (1) coupled K-Cl cotransport; (2) parallel activation of K^+^/H^+^ and Cl^−^/HCO_3_^−^ exchange; (3) parallel activation of K^+^ and Cl^−^ channels. The results of the present study provide firm evidence that thymocytes regulate their volume by the third mechanism. We believe that in earlier reports using radiolabeled isotopes [12,15], the K^+^ and Cl^−^ fluxes were apparently coupled due to the necessity to keep electroneutrality for the net ionic fluxes to take place: net movement of potassium ions is possible only when it is accompanied by an equivalent movement of chloride. Pharmacological studies of coupled K/Cl fluxes might be biased by the sensitivity of the VSOR Cl^−^ channels to the only available inhibitor of K-Cl cotransport, DIOA. Reported massive DIOA-sensitive Mg^2+^-efflux from thymocytes at high [K^+^] [42] could also be affected by the sensitivity of the VSOR Cl^−^ channel to this substance.

Thymus atrophy and thymocyte depletion are known to occur during infectious diseases and malnutrition and leads to immune deficiency [43]. The results of the present study suggest that the thymic VSOR Cl^−^ channel, as a major regulator of proliferation and apoptosis, may serve as a prospective target for immunomodulator drug discovery. The maxi-anion channel, as an important pathway for ATP and glutamate release in cell-to-cell signaling [8,9,18,44,45], may also represent a prospective target for immunotherapy.
